# Microvascular damage in diabetic nephropathy and the subsequent risk of Alzheimer’s disease: a systematic review

**DOI:** 10.1097/MS9.0000000000005443

**Published:** 2026-08-21

**Authors:** Noura Mohammed, Tho Alyazan Khalil Taher Al-Jabali, Nuha Yassien, Iman Osman Abufatima, Fatima Omer Ibrahim Ahmed, Soukina El Yazal, Wehiba Satti, Nadine Alalamat, Mohammed Aamer Abkar Ibrahim, Basma Tirelbar, Leepy Paudel, Islam Abdalla Abdelsadig Mahmoud, Leama Humdi, Hajer Ali, Mohammed Hammad Jaber Amin, Nadir Abdelrahman

**Affiliations:** a Faculty of Medicine, Karary University, Khartoum, Sudan; b Faculty of Medicine, University of Science and Technology, Khartoum, Sudan; c Faculty of Medicine, University of Juba, Juba, South Sudan; d Faculty of Medicine, University of Medical Sciences and Technology, Khartoum, Sudan; e Faculty of Medicine, Capital health Regional Medical Center, Trenton, New Jersey, USA; f Faculty of Medicine, University of Khartoum, Khartoum, Sudan; g Faculty of Medicine, Mutah University, Karak, Jordan; h Faculty of Medicine, Menofia University, Shebin El-Koum, Egypt; i Faculty of Medicine, Johns Hopkins University, Baltimore, Maryland, USA; j Faculty of Medicine, University of Kordofan, Kordofan, Sudan; k Faculty of Medicine, Zamzam University, Khartoum, Sudan; l Faculty of Medicine, Alzaiem Alazhari University, Kharotum, Sudan; m Faculty of Medicine, The Tele-Geriatric Research Fellowship, Michigan, USA

**Keywords:** Alzheimer’s disease, cognitive decline, diabetic nephropathy, kidney–brain axis, microvascular dysfunction

## Abstract

**Introduction:**

Diabetic nephropathy (DN), a major microvascular complication of diabetes, is increasingly implicated in systemic vascular dysfunction and may contribute to the pathogenesis of Alzheimer’s disease (AD). Microvascular injury is a recognized mechanism in AD, and DN-related damage may raise the risk of cognitive decline.

**Methods:**

Following the PRISMA 2020 guidelines, we searched PubMed, Scopus, and the Cochrane Library (2000–2025) for studies on DN-related vascular damage and cognitive outcomes. Quality was assessed using the Newcastle–Ottawa Scale and the AXIS tool.

**Results:**

Of 275 records, nine studies were included (cross-sectional and longitudinal, with up to 37.5 years of follow-up). DN was measured via eGFR, albuminuria, the fibrinogen-to-albumin ratio, or ICD codes; cognition via assessed using amyloid PET, neurocognitive tests, or registry data. All studies found that impaired kidney function was associated with a greater risk of AD or cognitive decline.

**Conclusion:**

Available evidence links DN-related microvascular damage to a higher risk of AD, suggesting a kidney–brain axis. However, causality remains uncertain and requires longitudinal and mechanistic studies to clarify these pathways.

## Introduction

Diabetic nephropathy (DN), one of the most common microvascular complications of diabetes mellitus, remains the leading cause of chronic kidney disease (CKD) and end-stage renal disease worldwide. Its prevalence continues to rise in parallel with the global diabetes epidemic, affecting approximately 30–40% of patients with type 1 or type 2 diabetes^[^1^]^. Traditionally regarded as a renal-specific disorder, DN is increasingly being recognized as a systemic condition with widespread vascular implications beyond the kidney.

HIGHLIGHTS

- Dn-related microvascular damage is linked to a higher risk of Alzheimer.
- Nine studies (2000–2025) showed consistent associations.
- Markers of kidney dysfunction were correlated with cognitive decline.
- Findings support a kidney–brain vascular connection.
- More longitudinal studies are needed to confirm causality.

Optimal management of DN focuses on strict glycemic and blood pressure control, along with the use of renin–angiotensin–aldosterone system inhibitors, to slow disease progression and reduce vascular complications^[^2^]^. Beyond the kidneys, diabetes frequently causes other microvascular injuries, including retinopathy and neuropathy, which share common pathogenic mechanisms such as chronic hyperglycemia, oxidative stress, and endothelial dysfunction^[^3^]^. These systemic microvascular changes may also contribute to cerebral small-vessel disease, linking diabetes-related vascular injury to an increased risk of cognitive decline and Alzheimer’s disease (AD)^[^4^]^.

AD, the most common cause of dementia, affects more than 55 million people globally, and its burden is projected to increase with an aging population^[^5^]^. While AD is primarily characterized by amyloid plaques, neurofibrillary tangles, and progressive cognitive decline, mounting evidence highlights the role of microvascular dysfunction in its pathogenesis^[^6^]^. Vascular contributions to cognitive impairment and dementia (VCID) have been extensively documented, and impaired microcirculation, endothelial dysfunction, and chronic inflammation are considered important risk factors for AD^[^7^]^. Microvascular damage in DN may also contribute to cerebral small-vessel injury, increasing susceptibility to vascular dementia^[^7^]^. This highlights the broader systemic impact of DN-related microvascular dysfunction.

Recent research has suggested that DN may be a risk factor for AD through shared microvascular pathways. Chronic hyperglycemia, oxidative stress, uremic toxin accumulation, and blood–brain barrier disruption are plausible mechanisms by which renal microvascular injury may contribute to neurodegenerative changes^[^8^]^. Observational cohort and cross-sectional studies have reported associations of reduced estimated glomerular filtration rate (eGFR) and albuminuria with an increased incidence of cognitive impairment or AD^[^6–8^]^. Moreover, biomarker studies have demonstrated correlations between impaired renal function and elevated amyloid and tau levels, supporting the hypothesis of a kidney–brain axis^[^9^]^.

Despite these emerging insights, the systemic relationship between DN-related microvascular damage and AD risk remains underexplored. Existing studies vary in design, diagnostic criteria, and methodological rigor, making it difficult to draw firm conclusions. Therefore, a comprehensive synthesis of the available literature is needed to clarify the extent and strength of this association, explore potential mechanistic pathways, and identify knowledge gaps to guide future research.

This systematic review aimed to evaluate the current evidence on the association between microvascular damage in DN and the risk of developing AD, assess the methodological quality of existing studies, and provide an integrated understanding of the kidney–brain connection, with a unique focus on DN-related microvascular injury as a distinct mechanistic pathway.

Titan Guidelines: This manuscript complies with the 2025 Titan Guidelines, declaring no use of AI^[^10^]^.

## Methods

### Study design

This systematic review was conducted in accordance with the Preferred Reporting Items for Systematic Reviews and Meta-Analyses (PRISMA) guidelines. This review was registered retrospectively with PROSPERO (ID: CRD420251140186). This review aimed to synthesize the existing evidence on the association between microvascular damage in DN and the subsequent risk of AD. The protocol for this review was developed prior to data collection, and all steps of the review process, including literature search, study selection, data extraction, and quality assessment, were performed systematically to minimize bias and ensure transparency.

### Information resources and search strategy

A comprehensive literature search was conducted to identify studies examining the relationship between DN, microvascular damage, and AD or dementia. The search was performed in PubMed, Scopus, and the Cochrane Library, and covered studies published between 2000 and 2025.

The following keywords and Medical Subject Headings (MeSH) were used in different combinations: *“diabetic nephropathy,” “diabetic kidney disease,” “microvascular damage,” “microvascular complications,” “microcirculation,” “Alzheimer’s disease,” “dementia,” “cognitive decline,”* and *“cognitive impairment.”* Boolean operators (AND, OR) were applied to combine terms appropriately.

Three separate strategies were applied to the PubMed search. The first strategy combined (“diabetic nephropathy” [tiab] OR “diabetic kidney disease” [tiab]) with (“Alzheimer’s disease” [tiab] OR dementia[tiab]), “Vascular dementia” [tiab] yielding 60 results. The second strategy combined (“diabetic nephropathy” [tiab] OR “diabetic kidney disease” [tiab]) with (“microvascular damage” [tiab] OR “microvascular complications”[tiab]) AND (“cohort study”[tiab] OR “case-control”[tiab] OR “observational study”[tiab]), yielding 90 results. The third strategy combined (“microvascular damage” [tiab] OR “microvascular complications” [tiab]) with (“Alzheimer’s disease”[tiab] OR dementia[tiab]) resulting in 103 studies.

In Scopus, the search was structured as follows: (TITLE-ABS-KEY(“diabetic nephropathy” OR “diabetic kidney disease” OR “diabetic nephropathies”)) AND (TITLE-ABS-KEY(“microcirculation” OR “microvascular damage” OR “microvascular injury” OR “microvascular complications”)) AND (TITLE-ABS-KEY(“Alzheimer’s disease” OR “cognitive decline” OR “cognitive impairment” OR “dementia”)), which yielded 20 results.

The Cochrane Library was also searched using the terms “microvascular damage,” “diabetic nephropathy,” and “Alzheimer’s disease,” yielding two results.

A total of 275 records were retrieved from all the databases. After duplicates were removed, the remaining studies were screened for relevance by two reviewers based on their titles and abstracts. The full texts of potentially eligible articles were then assessed against inclusion and exclusion criteria, with any disagreements resolved through discussion or, when necessary, consultation with a third reviewer.

### Inclusion criteria

Studies were considered eligible for inclusion if they were peer-reviewed original research articles published in English, conducted in human populations, and involving adult participants aged 18 years or older. The eligible designs included observational studies (cohort, case–control, and cross-sectional), longitudinal studies, and clinical trials. To be included, studies had to assess microvascular damage in the context of DN and evaluate cognitive outcomes or diagnoses consistent with AD. Only studies published in English were included.

Studies were excluded if they involved non-human subjects or animal models, did not specifically examine both DN and AD (or related cognitive impairment), or were not published in English. Reviews, editorials, commentaries, conference abstracts, and case reports were also excluded. Additionally, studies with insufficient data were excluded.

### Data extraction and management

Data were extracted using a standardized form developed for this review. Two reviewers independently extracted relevant information from each included study, including study design, sample size, participant demographics, methods used to assess microvascular damage in DN, measures of cognitive outcomes related to AD, and reported confounding factors. Any discrepancies between reviewers were resolved through discussion, and, when consensus could not be reached, a third reviewer adjudicated.

### Quality assessment and risk of bias

The methodological quality and risk of bias of the included studies were independently appraised by three reviewers. The Newcastle–Ottawa Scale (NOS) was applied to observational cohort and case-control studies^[^11^]^, while cross-sectional studies were evaluated using the AXIS tool^[^12^]^. Systematic reviews were assessed using AMSTAR 2^[^13^]^. Each tool was applied according to established guidelines, with reviewers independently scoring each study. Discrepancies were resolved through discussion, and a final consensus score was assigned. Inter-rater reliability was monitored throughout the process and achieved substantial agreement (Cohen’s kappa = 0.78). For this review, studies were classified according to their total quality score: high quality if they met ≥75% of criteria (e.g., NOS ≥ 7 stars; AXIS ≥ 15/20 items), moderate quality if they met 50–74% of criteria (e.g., NOS 5–6 stars; AXIS 10–14 items), and low quality if they met <50% of criteria (e.g., NOS ≤ 4 stars; AXIS < 10 items). This approach ensured consistent and transparent interpretation across study designs and facilitated consideration of methodological rigor in the synthesis.

### Data synthesis

Given the heterogeneity in study designs, populations, and outcome measures, a qualitative synthesis was employed. Findings were summarized with emphasis on the consistency of the associations between microvascular damage in DN and the risk of AD. Patterns were also evaluated according to study type, methodological quality, and risk-of-bias rating to provide a comprehensive interpretation of the evidence.

## Results

### Study selection

A total of 275 records were retrieved from the following electronic databases: PubMed (*n* = 253), Scopus (*n* = 20), and the Cochrane Library (*n* = 2). After removing 17 duplicates were removed, 258 unique records remained for screening. Of these, 258 records underwent title and abstract screening, and 232 were excluded because they did not meet the eligibility criteria. Twenty-six full-text articles were then evaluated, and none were excluded because of retrieval problems.

**Figure 1.**
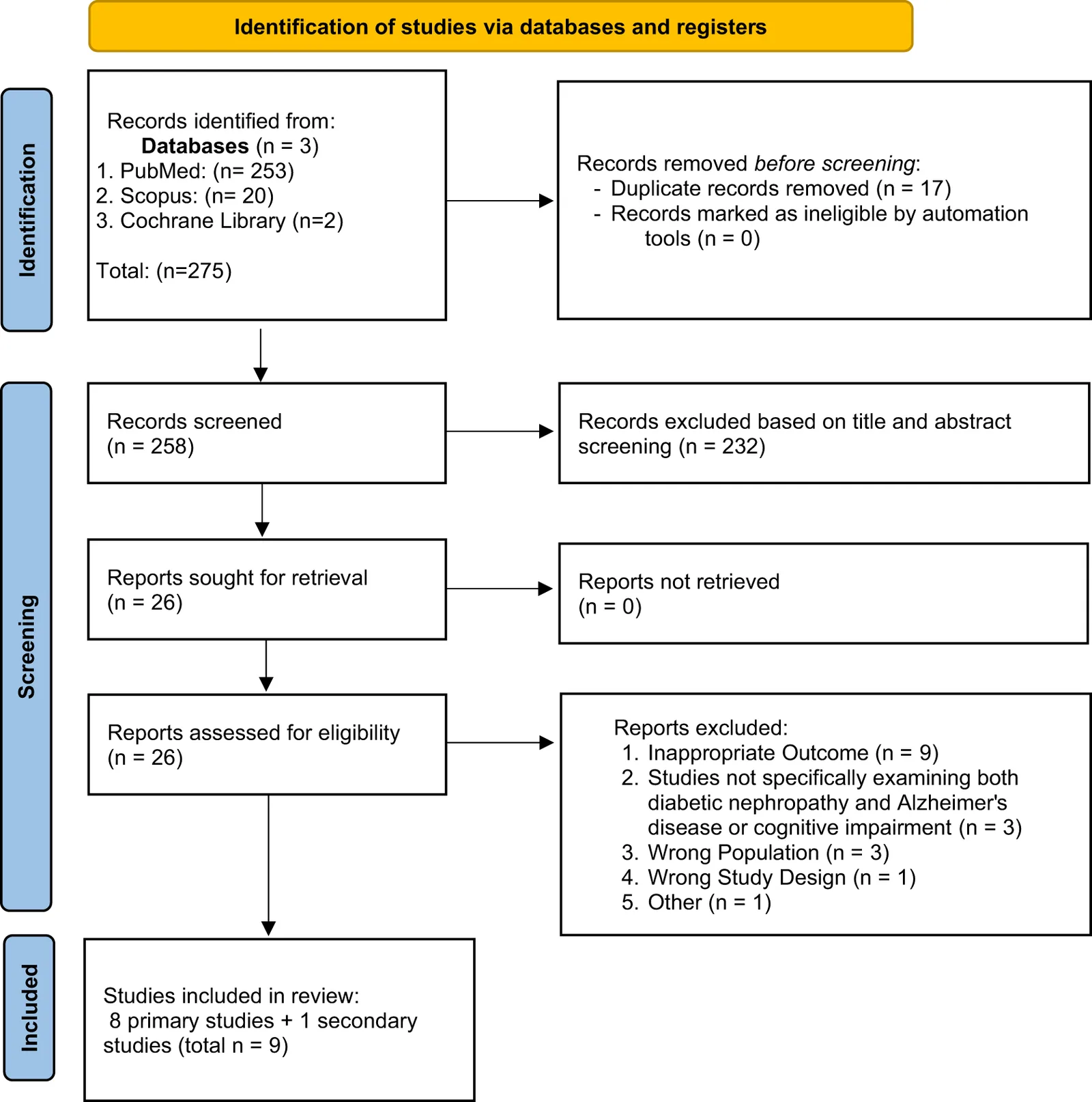
PRISMA flow diagram of included studies.

During the full-text assessment, studies were excluded for the following reasons: inappropriate outcomes (*n* = 9; e.g., reporting only general cognitive function or renal biomarkers without neurocognitive assessment), not addressing both DN and either AD or cognitive impairment (*n* = 3; these studies examined only one of the two conditions despite a broad search), irrelevant populations (*n* = 3; e.g., pediatric or non-diabetic cohorts), wrong study design (*n* = 1; narrative review), and other reasons (*n* = 1; insufficient methodological detail). Nine studies were included in the review, consisting of eight primary investigations and one secondary study. The complete selection process is presented in the PRISMA 2020 flow diagram (Fig. 1).

### Baseline characteristics of the studies

Table 1 summarizes the baseline characteristics of the included studies. Five observational studies published between 2018 and 2025 were included, featuring sample sizes ranging from small cohorts (e.g., 216–328 participants in Chinese populations) to large national registry studies with tens of thousands to more than a million individuals (e.g., Taiwan and Finland). This variation in sample size influences both statistical power and generalizability.

**Table 1:** Baseline characteristics of the studies.

Study ID	Study design	Sample size	Country/setting	Funding source	Age (Mean ± SD)	Sex (% female)	Diabetes type	DN definition	AD definition	
Arslan *et al*^[^9^]^	Observational cohort	242	Canada/Sweden	Multiple academic, governmental, and philanthropic organizations (see “Sources of funding” section)	70.3 (Cognitively unimpaired, IQR: 65.7–74.7)	66.40%	Not specified	Not applicable	Defined via amyloid PET positivity using [18 F]-AZD4694 SUVR >1.55	
					72.2 (Mild cognitive impairment, IQR: 66.3–75.9)					
					66.9 (Alzheimer’s disease, IQR: 61.1–73.5)					
					64.0 (Non-AD Dementia, IQR: 57.5–69.6)					
Yen *et al*^[^14^]^	Nationwide retrospective cohort study	1 212 966 initially identified; 568 577 without microvascular disease, 79 241 with microvascular disease after exclusions	Taiwan	Taiwan Ministry of Health and Welfare, MOST Clinical Trial Consortium	~50–80 +	–	T2DM	Diabetic neuropathy (DN) diagnosed via ICD codes within 1 year of T2D diagnosis	Alzheimer’s disease (AD) diagnosed via ICD codes and dementia medications (≥2 outpatient visits or 1 hospitalization)	
Satuli-Autere *et al*^[^15^]^	Nationwide observational cohort study	4 261 individuals (T1D); 11 653 matched controls without diabetes	Finland	Folkhälsan Research Foundation, Wilhelm and Else Stockmann Foundation, Finnish Diabetes Research Foundation, Sigrid Jusélius Foundation, others	37.4 years (IQR: 28.7, 46.6)	47.80%	T1DM	DKD defined as albuminuria or kidney replacement therapy; severe retinopathy (retinal photocoagulation); cardiovascular disease (CVD) history	(AD) via ICD codes (ICD-8/9: 3310; ICD-10: G30/F00)	
Li *et al*^[^16^]^	Retrospective cross-sectional study	328 inpatients with T2DM	China	National Natural Science Foundation of Heilongjiang Province, Heilongjiang Health Committee, others	52.62 ± 10.92	38.10%	T2DM	Not explicitly defined	Mild cognitive impairment (MCI) diagnosed via MoCA score <26 (Montreal Cognitive Assessment)	
Li *et al* ^[^17^]^	Multicenter retrospective study	216 patients with T2DM	China	No specific funding	NC: 53.3 ± 15.52	50%	T2DM	Early T2DN: UAER 20–200 μg/min	Cognitive impairment assessed via MMSE (≤26) and MoCA (≤26)	
								Clinical T2DN: UAER ≥200 μg/min		
					DW (T2DM without nephropathy): 53.3 ± 15.52					
					DN-III (early T2DN): 55.1 ± 14.49					
					DN-IV (clinical T2DN): 54.1 ± 15.84					
Fang *et al* ^[^18^]^	cross-sectional observational study	66 total with 40 T2DM, 26 healthy volunteers	China/Shanghai General Hospital	Shanghai Pujiang Program (15PJD032) and Shanghai Municipal Education Commission-Gaofeng Clinical Medicine Grant (20152231)	43 ± 9.7	61.50%	T2DM	Defined by microalbuminuria as a urinary albumin-to-creatinine ratio (UACR) > 30 μg/mg on two consecutive days.	patients were *dementia-free*, screened using MMSE = 30 criterion (excluded if signs of dementia)	
Umegaki *et al*^[^19^]^	Longitudinal cohort study (6-year follow-up)	261	Japan/Multiple sites including Tokyo Metropolitan Geriatric Hospital and University of Nagoya	Research Grants for Longevity Sciences from the Ministry of Health, Labour and Welfare (H12-, H15-, H17-Choju series) + Japan Foundation for Aging and Health	70.6 ± 4.3	57.50%	T2DM	Defined by urinary albumin-to-creatinine ratio (ACR); nephropathy = ACR ≥30 mg/g	not directly defined	
Gul *et al*^[^20^]^	Clinical cross-sectional	148 total with 104 with T2DM and 44 nondiabetic control	Turkey, Uludag University Medical School, Bursa	None declared	65.86 ± 5.19	59.60%	T2DM	Defined by microalbuminuria defined as urinary microalbumin/creatinine ratio (MCr) > 30 mg/mg in spot urine	Cognitive function assessed using the Standardized Mini Mental State Examination (SMMSE)	

The majority of studies have focused on type 2 diabetes, whereas a single study examined type 1 diabetes. Definitions of DN and AD also differed, using criteria such as albuminuria, a decline in eGFR, ICD coding, amyloid PET scans, or cognitive assessment tools.

### Comprehensive summary

Five studies were included in this qualitative analysis (Table 2). Two studies were cross-sectional, while three had longitudinal follow-up periods ranging from 67 months to 37.5 years. The assessment of DN varied, including plasma creatinine and eGFR, ICD codes, clinical evaluation of albuminuria and retinopathy, fibrinogen-to-albumin ratio, and urinary albumin excretion rate.

**Table 2:** Summary of study methodologies and assessments.

Study ID	Primary objective	Follow-up	DN assessment	AD/MCI assessment	Key biomarkers	Statistical analysis
Arslan *et al*^[^9^]^	Kidney function & plasma AD biomarkers	Cross-sectional	eGFR, creatinine	Amyloid PET	Aβ species, p-tau, GFAP, NfL	Correlation, regression, AUC
Yen *et al*^[^14^]^	Microvascular disease & dementia risk in T2D	6.54 years	ICD codes	ICD codes + medications	Not reported	Cox models, Kaplan-Meier
Satuli-Autere *et al*^[^15^]^	Neurodegenerative disease risk in T1D	37.5 years	Albuminuria, retinopathy, CVD	National registers	HbA1c, eGFR, albuminuria	SIRs, competing risk
Li *et al*^[^16^]^	FAR association with MCI in T2D	Cross-sectional	Fibrinogen-to-albumin ratio	MoCA <26	Fibrinogen, albumin	Logistic regression, ROC
Jinyu Li *et al*^[^17^]^	Cognitive impairment & renal disease in T2D	67 months	UAER, eGFR	MMSE, MoCA	UAER, HbA1c, cerebral metabolites	Correlation, regression
Umegaki *et al*^[^19^]^	Brain volume predictors of AD in MCI	24 months	Urinary ACR	Clinical criteria	Brain volumes	Cox model, ROC
Gul *et al*^[^20^]^	Glycemic control, microalbuminuria & cognition	6 years	Urinary ACR	MMSE decline	HbA1c, lipids, albumin	Regression models

Aβ, amyloid-beta; p-tau, phosphorylated tau; GFAP, glial fibrillary acidic protein; NfL, neurofilament light chain; eGFR, estimated glomerular filtration rate; PET, positron emission tomography; AUC, area under the curve; ICD, International Classification of Diseases; CVD, cardiovascular disease; SIRs, standardized incidence ratios; HbA1c, glycated hemoglobin; FAR, fibrinogen-to-albumin ratio; MCI, mild cognitive impairment; MoCA, Montreal Cognitive Assessment; ROC, receiver operating characteristic; UAER, urinary albumin excretion rate; MMSE, Mini-Mental State Examination; ACR, albumin-to-creatinine ratio.

Cognitive outcomes were evaluated using amyloid PET imaging, health registries, hospital and outpatient records, and standardized neurocognitive tests (MMSE and MoCA). Several biomarkers have been investigated, including Aβ isoforms, phosphorylated tau, GFAP, neurofilament light chain, HbA1c, and fibrinogen. Statistical approaches ranged from correlation analyses (Spearman and Pearson) to more advanced methods, including multivariable regression, standardized incidence ratios, and Cox regression models. Overall, all studies reported associations between impaired kidney function and an increased risk of AD or cognitive decline (Table 3).

**Table 3:** Comprehensive summary of study outcomes.

Study ID	Main outcome(s)	DN–AD association	Significant findings	Subgroup analyses	Mechanistic pathways	Limitations
Arslan *et al*^[^9^]^	Lower eGFR vs. AD biomarkers	Yes	↓ eGFR → ↑ Aβ42, Aβ40, NfL, GFAP	CKD stages 1–3; amyloid + vs.−	Impaired biomarker clearance; stronger for NfL	Mild CKD; cross-sectional; limited diversity
Yen *et al*^14^^]^	Dementia risk by microvascular disease	Yes	DN ↑ risk of AD, VaD, all-cause dementia	DKD strongest for VaD	Inflammation, glycation, oxidative stress	ICD bias; no lifestyle data; Taiwan-only
Satuli-Autere *et al*^[^15^]^	Neurodegenerative disease incidence	Yes	DKD ↑ AD & dementia (SIR 3–5); CVD ↑ AD	Childhood vs. adult T1D	Chronic inflammation, vascular damage	Registry bias; missing DN data
Li *et al*^[^16^]^	FAR and MCI/MoCA	Yes	↑ FAR → ↑ MCI (OR 34.7); ↓ MoCA (*r* = −0.55)	By age (<60 vs. ≥60)	Inflammation, oxidative stress	Cross-sectional; single center
Li *et al*^[^17^]^	UAER/eGFR and cognition	Yes	↑ UAER, ↓ eGFR → ↓ MoCA	DN stages III–IV vs. DW	Microvascular damage, cholinergic imbalance	Retrospective; small sample
Umegaki *et al*^[^19^]^	Cognitive decline (6 years, MMSE)	Yes	DN predicted ≥5-point MMSE drop	DN vs. no-DN	Endothelial dysfunction, microvascular injury	MMSE only; confounding; small sample
Gul *et al*^[^20^]^	Microalbuminuria and cognition	No	No difference in SMMSE by albuminuria	Normo vs. microalbuminuria	Vascular hypothesis	Small sample; no imaging data

Aβ, amyloid-beta; CKD, chronic kidney disease; CVD, cardiovascular disease; DKD, diabetic kidney disease; DN, diabetic nephropathy; DW, diabetes without nephropathy; eGFR, estimated glomerular filtration rate; FAR, fibrinogen-to-albumin ratio; GFAP, glial fibrillary acidic protein; HbA1c, glycated hemoglobin; ICD, International Classification of Diseases; MCI, mild cognitive impairment; MMSE, Mini-Mental State Examination; MoCA, Montreal Cognitive Assessment; NAA, N-acetylaspartate; NfL, neurofilament light chain; OR, odds ratio; SIR, standardized incidence ratio; SMMSE, Standardized Mini-Mental State Examination; T1D, type 1 diabetes; UAER, urinary albumin excretion rate; VaD, vascular dementia.

### Quality assessment results

The methodological quality of the included studies was assessed using the NOS for cohort studies and the AXIS tool for cross-sectional studies. Overall, the studies were of moderate to high quality. Five studies received a high NOS score of 8 or 9 out of 9, reflecting strengths such as large representative sample sizes, long-term follow-up, reliable registry-based data, and robust adjustment for key confounders, including age and sex. The cross-sectional studies, evaluated using the AXIS tool, were rated as being of moderate quality. Although they possessed clear aims, appropriate designs, and validated measurement tools, common limitations were noted.

These limitations include a lack of statistical justification for the sample size, insufficient detail on participant recruitment, and restricted generalizability due to single-center designs or selected inpatient populations. The most frequent limitations across all studies were the potential for residual confounding from unmeasured variables (e.g., lifestyle or genetic factors) and the inherent constraints of observational designs, which preclude causal inference.

## Discussion

This systematic review provides evidence that microvascular damage in DN is associated with an increased risk of AD and cognitive decline. All the included studies, regardless of design, demonstrated consistent associations between impaired renal function and neurocognitive outcomes. These findings are biologically plausible, as DN and AD share overlapping mechanisms, including endothelial dysfunction, accumulation of advanced glycation end-products, oxidative stress, and low-grade inflammation^[^21–23^]^. The observed links with AD-related biomarkers, such as Aβ isoforms, phosphorylated tau, and neurofilament light chain, further support a mechanistic connection between renal microvascular injury and neurodegeneration^[^24–26^]^.

Our review extends prior knowledge on diabetes and dementia risk by emphasizing the specific contributions of DN. Mechanistic evidence supports the observed link between DN and AD. Albuminuria reflects systemic endothelial dysfunction and is associated with cerebral small vessel disease. Oxidative stress, inflammation, blood–brain barrier disruption, and impaired amyloid beta clearance may further drive neurodegeneration, providing a biological rationale for how renal microvascular injury could contribute to cognitive decline. Previous meta-analyses have shown a two-fold higher risk of dementia in individuals with diabetes; however, few have distinguished the impact of microvascular complications^[^27,28^]^. The studies included here suggest that DN may act not only as a complication of diabetes but also as a mediator of cognitive decline. Importantly, these associations were observed across a range of populations, from smaller clinical cohorts in China to large-scale registry studies in Taiwan and Finland, enhancing external validity. Although most data were derived from type 2 diabetes populations, preliminary evidence in type 1 diabetes suggests that this relationship may be broader^[^29^]^.

The clinical implications of our findings are significant. Therefore, DN may serve as a readily identifiable marker for stratifying the risk of dementia in patients with diabetes. Incorporating cognitive assessments into the routine management of individuals with renal impairment could enable earlier recognition of cognitive decline^[^30^]^. Furthermore, therapies that slow the progression of DN, such as strict glycemic, blood pressure, and lipid control, may yield downstream benefits in reducing dementia risk^[^31–33^]^. Exploration of DN-related biomarkers as surrogate indicators of early neurodegenerative processes could further refine risk prediction and preventive strategies^[^34^]^.

However, heterogeneity across studies should be acknowledged. Differences in study populations (age, sex, ethnicity), diabetes type (type 1 vs. type 2), definitions of DN (e.g., albuminuria, eGFR thresholds), cognitive or AD assessment tools, and the length of follow-up limit direct comparability. This variability may influence the observed strength and consistency of associations and precludes formal meta-analysis in most cases. A quantitative assessment of heterogeneity was not feasible due to these differences, highlighting the need for future longitudinal studies with standardized DN and cognitive outcomes, as well as comprehensive adjustment for potential confounders, to more accurately define the relationship between DN-related microvascular damage and risk of AD.

### Limitations

Despite the compelling associations observed, several limitations must be acknowledged. Reverse causation cannot be excluded, particularly in cross-sectional studies, and residual confounding from unmeasured factors such as lifestyle, comorbidities, or genetic risk (e.g., APOE genotype) may influence results. Publication bias is possible, as studies reporting null associations may be underrepresented. Outcome definitions were heterogeneous, with DN measured by albuminuria, eGFR decline, or clinical coding, and with cognitive outcomes ranging from clinical dementia diagnoses to cognitive screening tests, while most studies did not directly assess AD pathology, such as amyloid or tau accumulation. Furthermore, the predominance of observational designs limits causal inference, and small, single-center studies may reduce generalizability. Nevertheless, the use of long follow-up periods and adjustment for major confounders strengthen confidence in the observed associations.

### Future directions

To advance understanding of the link between DN and AD, future research should prioritize longitudinal cohort studies that can better establish temporality and causal pathways. Incorporation of neuroimaging and fluid biomarkers – including amyloid, tau, and markers of cerebrovascular injury – will be critical for clarifying mechanistic connections between renal microvascular damage and neurodegeneration. Randomized controlled trials testing whether interventions that slow DN progression or protect the microvasculature (e.g., strict glycemic, blood pressure, and lipid control or novel renoprotective therapies) can reduce AD risk would provide the strongest evidence of causality. Expanding research to include type 1 diabetes populations and underrepresented ethnic groups will improve generalizability, while harmonized definitions of DN and cognitive outcomes will facilitate cross-study comparisons and meta-analyses. Addressing these gaps will be key to translating mechanistic insights into strategies for preventing cognitive decline in patients with diabetes.

## Clinical implications

The findings of this review suggest that DN may serve as an early clinical marker to identify individuals at increased risk of cognitive decline and AD. Incorporating routine assessment of renal function, such as albuminuria and estimated glomerular filtration rate, into diabetes management could help stratify patients for closer cognitive monitoring. Interventions that slow the progression of DN, including strict glycemic control, blood pressure management, lipid control, and therapies such as renin–angiotensin system blockade or sodium-glucose cotransporter 2 inhibitors, may have downstream benefits in preserving cognitive function. Early recognition and management of DN could therefore represent a practical strategy to reduce the dual burden of diabetes and dementia.

## Conclusion

Only a limited number of studies – predominantly cross-sectional – have directly examined the link between DN and AD; therefore, the strength of causal conclusions must be interpreted cautiously. While DN may serve as a biomarker of systemic microvascular damage, directly attributing increased AD risk to DN requires longitudinal studies that adequately control for confounders such as hypertension, dyslipidemia, glycemic control, and APOE genotype, which are often lacking. Nevertheless, this systematic review highlights consistent evidence that microvascular damage in DN is associated with an elevated risk of cognitive decline and AD. DN may therefore act both as a clinical marker and as a potential mechanistic contributor to neurodegeneration in diabetes, suggesting that early identification and optimal management of DN could help mitigate the dual burden of diabetes and dementia.

## Data Availability

All data analyzed during this study are included in this published article.

## Research registration unique identifying number (UIN)

PROSPERO ID: CRD420251140186.
